# Introduction to ‘Synthesis and chemical biology of macrocycles’

**DOI:** 10.1039/d2cb90018a

**Published:** 2022-07-18

**Authors:** Gong Chen, Monika Raj, Andrei Yudin

**Affiliations:** State Key Laboratory of Elemento-Organic Chemistry, Nankai University Tianjin 300071 China; Department of Chemistry, Emory University 1515 Dickey Drive Atlanta, GA 30322 USA; Davenport Chemistry Laboratories, Department of Chemistry, University of Toronto 80 St. George Street Toronto Ontario M5S 3H6 Canada andrei.yudin@utoronto.ca

## Abstract

Gong Chen, Monika Raj and Andrei Yudin introduce the *RSC Chemical Biology* themed collection on the ‘Synthesis and chemical biology of macrocycles’.
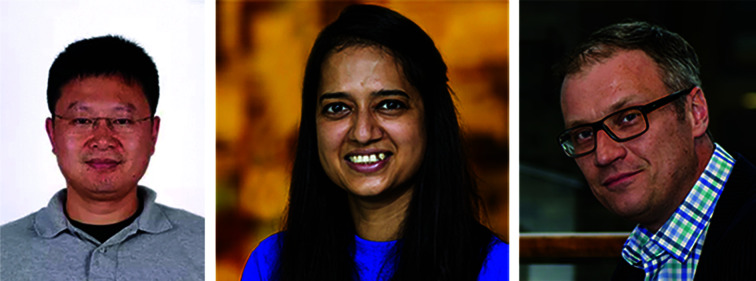

This themed collection offers a collection of articles that showcase the ongoing interest in macrocycles. Recent years have witnessed the development of new synthetic and biological strategies to construct large rings composed of amino acid residues. In addition, significant progress has been made in efforts to understand and predict the properties of macrocycles. We have collected contributions that are representative of the tremendous promise and pace of growth in this area.

As part of this collection, Hackenberger and colleagues describe an mRNA-based display technique to screen a library of *in vitro*-translated cyclic peptides (https://doi.org/10.1039/D1CB00056J). As a result, the authors have identified and characterized a macrocyclic ligand with picomolar potency against MDM2. The contribution from Li's lab documents a stapled peptide inhibitor of the PSD-95 GK domain that has led to a 25-fold increase in the binding affinity (https://doi.org/10.1039/D1CB00087J). This study underscores that increasing the stability of stapled peptides can be an effective strategy for the rational design of protein–protein interaction inhibitors. Kennedy and co-workers continue the theme of stapled peptides and report the development of two cell-permeable hydrocarbon-stapled molecules that bind PKC to disrupt PKC–gravin complex formation in cells (https://doi.org/10.1039/D1CB00106J). The contribution from the Chen lab discusses cyclic peptides that have been approved for clinical use in the past two decades (https://doi.org/10.1039/D1CB00154J). This paper further highlights the challenges and opportunities in the development of macrocyclic drugs. Meanwhile, Suga's lab documents how genetic code reprogramming has enabled them to ribosomally synthesize various AIP-I analogs (https://doi.org/10.1039/D1CB00225B). This study nicely reveals how amino acid substitution in the thiolactone fragment drastically alters the resistance to the promotion of the S-to-O acyl transfer. Petersson and colleagues have performed a systematic investigation of the impact of thioamide incorporation in a β-hairpin scaffold, which offers insights regarding the position of thioamide incorporation (https://doi.org/10.1039/D1CB00229E). The paper from Unsworth's lab documents a novel conjugate addition/ring expansion (CARE) cascade reaction sequence for the synthesis of medium-sized and macrocyclic bis-lactams from primary amines and cyclic imides (https://doi.org/10.1039/D1CB00245G). Lastly, the Yudin lab's contribution introduces “higher-order” *φ*/*ψ* plots, termed macrocycle conformational maps (MCMs), as a tool for evaluating and comparing the conformations of macrocycles (https://doi.org/10.1039/D2CB00016D).

Taken together, the papers collected in this themed collection represent the state of the art in macrocycles and we hope that our readers will find these contributions equally inspiring and thought-provoking.

## Supplementary Material

